# Deciphering Cryptic Population Structure in Western Sandhill Crane Subspecies (*Antigone canadensis*) of the Pacific Flyway

**DOI:** 10.1002/ece3.71475

**Published:** 2025-05-28

**Authors:** Ruth Joy, Krista Roessingh, Kathleen Meszaros, Allyson Miscampbell, Carol Ritland, Matt Hayes, Gary Ivey, Mike Petrula, Jeffrey B. Joy

**Affiliations:** ^1^ School of Environmental Science Simon Fraser University Burnaby British Columbia Canada; ^2^ Citizen Scientist Denny Island British Columbia Canada; ^3^ Genetic Data Centre, Faculty of Forestry University of British Columbia Vancouver British Columbia Canada; ^4^ Illinois Audubon Society Springfield Illinois USA; ^5^ International Crane Foundation Bend Oregon USA; ^6^ Alaska Department of Fish and Game, Division of Wildlife Conservation Waterfowl Program Anchorage Alaska USA; ^7^ Molecular Epidemiology and Evolutionary Genetics BC Centre for Excellence in HIV/AIDS Vancouver British Columbia Canada; ^8^ Bioinformatics Programme University of British Columbia Vancouver British Columbia Canada; ^9^ Division of Infectious Diseases, Department of Medicine University of British Columbia Vancouver British Columbia Canada

**Keywords:** gene flow, non‐invasive sampling, Pacific Flyway, population genetics, population management, Sandhill Cranes

## Abstract

Population segregation catalyses genetic differentiation and can lead to speciation. Population genetic structure is also critically important for population management, especially in species characterised by small, isolated populations. Sandhill Crane (*Antigone canadensis*) populations of the Pacific Flyway are made up of breeding populations nesting west of the Rocky Mountains, and isolated by intermediate mountain ranges. Current management policy in British Columbia treats all Sandhill Cranes as a single population, whereas in the western United States, subpopulations are subject to population‐specific management. Here, we analyse microsatellite markers, mitochondrial DNA sequences, and mitochondrial haplogroups derived from 203 individual Sandhill Cranes to elucidate the population genetic structure of cranes migrating along the Pacific Flyway to summer breeding habitat on the North and Central Coast of British Columbia and Southeast Alaska. STRUCTURE, AMOVA, *F*_ST_, DAPC, and phylogenetic analyses reveal that geographically separated crane populations along the west coast of North America show substantial genetic differentiation in the Pacific Flyway. These findings are consistent with behavioural and ecological evidence—divergent diets, flyways, and breeding habitats. We conclude that the relatively small coastal Sandhill Crane populations deserve special management consideration to safeguard their genetic diversity and adaptations, and to mitigate deleterious impacts of current and future climate change scenarios.

## Graphical abstract

Sandhill Crane populations of the Pacific Flyway are made up of breeding populations nesting west of the Rocky Mountains, and isolated by intermediate mountain ranges. Here, we analyze microsatellite markers, mitochondrial DNA sequences, and mitochondrial haplogroups to elucidate population genetic structure and found substantial genetic differentiation. The conservation of Sandhill Cranes west of the Rocky Mountains depends on safeguarding the genetic diversity and adaptations within and between populations to protect them from current and future climate scenarios.

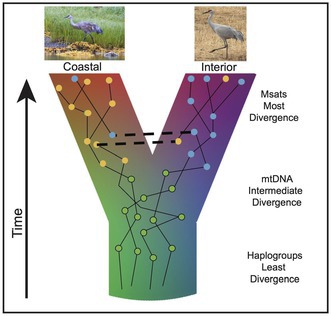

## 1 Introduction

A clear understanding of population genetic diversity and its effects on population genetic structure can be critically important for species management plans. Maintenance of intra‐species genetic diversity facilitates species abilities to adapt to changing habitats and selective environments and thus should be a key goal of conservation and management planning (Sgrò et al. 2011). Population genetic structure depends on a variety of factors, including differential reproductive success and dispersal and settling patterns on breeding areas. For migratory species, these factors can be compounded by long‐distance movements and intermixing of different genetic groups on staging and wintering areas (Faaborg et al. 2010). Significant population genetic structure results from reproductive isolation due to physical barriers such as geographic separation or behavioral barriers directed by natural or sexual selection. Given enough time, reproductive isolation can also result from genetic drift (Turbek et al. 2018). Genetic differentiation leading to reproductive isolation between allopatric breeding populations may result from selection, genetic drift, or their combination (Turbek et al. 2018). Determining the context of divergence and extent of gene flow among populations of migratory species is important for understanding the evolutionary trajectory of populations and, critically, for assigning conservation priority (Jetz et al. 2014; Stein et al. 2018).

Sandhill Cranes (*Antigone canadensis*) comprise a complex of geographically separated breeding populations with varying levels of conservation importance tied to national, state, and provincial conservation priorities. This charismatic species is known for its elaborate courtship displays that include ritualized dancing and synchronized calls, useful for both establishing and maintaining pair bonds. Sandhill Cranes form long‐term pair bonds, often staying together year‐round. They display high fidelity to breeding territories and established migratory flyways (Walkinshaw 1973). Furthermore, young cranes display strong philopatry (Walkinshaw 1949; Littlefield and Ivey 1995). However, some birds show punctuated long‐distance dispersal, especially at young ages (Hayes 2015). These life history properties, coupled with specific habitat requirements (nesting in a variety of freshwater wetland types and feeding in various upland habitats) determine broad‐scale genetic patterns of differentiation.

Six morphological and geographic subspecies of Sandhill Cranes have been described across their breeding distribution (Mirande and Harris 2019), from Siberia in Eastern Asia through North America to Cuba in the Caribbean. The three migratory subspecies have distributions that roughly follow a north–south ecotone with *A.c. canadensis* (‘Lesser’) breeding in the arctic, *A.c. rowani* (‘Canadian’) in the subarctic and the Pacific coast, and *A.c. tabida* (‘Greater’) in the south (Littlefield and Ivey 2002), and all are distinguished by morphology (bill size and body length) and geography (Johnsgard 1983). The validity of *A.c. rowani* as a genetically separate subspecies has been debated by several studies (Rhymer et al. 2001; Glenn et al. 2002; Petersen et al. 2003, and Jones et al. 2005) suggesting that its morphological differences may be only clinal, however these studies omitted samples from cranes breeding west of the Rocky Mountain range.

Sandhill Cranes that migrate within the Pacific Flyway through British Columbia (BC) and Alaska for the most part share wintering grounds in California's Central Valley, but during migration and breeding are split between the ‘interior’ migratory flyway and the ‘coastal’ flyway. The ‘interior’ migration corridor includes cranes that migrate east of the Cascade and Coast Mountains, members of the Central Valley Population (> 8600 cranes, mostly *A.c. tabida*) that breed from Northern California to central BC (Pacific Flyway Council 1997; Littlefield and Ivey 2002), as well as members of the Pacific Coast Population (> 36,100 cranes, mostly *A.c. canadensis* and a smaller number of *A.c. rowani*) that breed in southwest Alaska (Caven 2023; Petrula and Rothe 2005). The Central Valley population prefers freshwater marsh or groundwater fens, grasslands, farm fields or grazing pastures, and are adapted to nesting and foraging in more terrestrial environments (Cooper 1996; Jones et al. 2005). Cranes that use the ‘coastal’ flyway are also members of the Pacific Coast Population, but they migrate up the west side of the Coast Mountains and breed from the north end of Vancouver Island to southeast Alaska, and are thought to be *A.c. rowani* (Ivey et al. 2005; Stinson 2017). Cranes from this coastal population utilise marine intertidal habitats, foraging on marine molluscs in estuaries and coastal beaches and nesting in bogs close to the ocean (Hearne and Hamel 2003; Roessingh 2012).

Prior to 2009, the BC government assigned all Sandhill Cranes elevated conservation status in the province, due to the paucity of information regarding their individual population status and potential threats to their breeding habitat (Cooper 1996). In 2009, the status changed to ‘apparently secure’, without differentiating between different sized populations nor conservation concerns related to breeding habitats and land use management. Delisting was due to widespread breeding records and a rise in overall population and range since 1970 (BC Conservation Data Centre 2018). Meanwhile, all Sandhill Cranes remain on the endangered list in Washington State (Washington Department of Fish and Wildlife 2021), and *A.c. tabida* is listed as ‘sensitive’ in Oregon State (Oregon Department of Fish and Wildlife 2016) and ‘threatened’ in the State of California (California Natural Diversity Database 2021), where the wintering habitat of BC's cranes is increasingly facing loss and encroachment, as well as impacts of land‐use and climate change.

Population genetic structure of cranes along the Pacific Flyway was quantified in Hayes (2015) using Amplified Fragment Length Polymorphism (AFLP) markers, mitochondrial DNA (mtDNA) haplotypes, and morphological data. Hayes (2015) found evidence of population genetic structure but showed some gene flow between coastal‐breeding birds and interior Central Valley cranes in Oregon and California (Hayes 2015). This analysis was restricted to eight samples from the coastal breeding population, and zero from interior BC. We set out to build on Hayes (2015) analysis to address the information deficit in the population genetic structure of the Pacific Flyway Sandhill Crane populations west of the Rocky Mountains of British Columbia and southern Alaska. Specifically, we test the hypothesis that gene flow restriction between BC coastal and interior breeding populations, two regions separated by the Pacific Mountain System (also known as the ‘Coast Mountains’), which includes the Cascade Range in the United States, has resulted in genetic structure among these populations. We predict genetic markers derived from samples from coastal breeding cranes will show significant divergence from genetic markers derived from cranes breeding in the Interior. To test this hypothesis, we analysed microsatellite markers and mtDNA control region sequences to compare Sandhill Cranes nesting across the region covering southwest to southeast Alaska, coastal and interior BC, mtDNA control region sequences were also grouped into haplogroups following Hayes (2015). We also sought to test the efficacy of microsatellite primers designed for different crane species in amplifying markers from Sandhill Cranes.

## 2 Methods

### 2.1 Specimen Collections

To compare between Sandhill Crane populations, we collected naturally‐shed feathers from cranes within their breeding territories. The four sample collection areas were (1) the ‘Coast’ population including the islands of the remote Central and North Coast of BC and Southeast Alaska, (2) the offshore archipelago of ‘Haida Gwaii’, (3) the intermountain ‘Interior BC’ grassland regions between the Coast Mountains to the west and the Rocky Mountains to the east, and (4) ‘Southwest Alaska’. Our field collection focused on naturally shed feathers, minimising impact and disturbance to the cranes, and allowing us to maximise volunteer participation, a strategy enabling sample collection over a broad geographic area (Segelbacher 2002). We used public outreach tools including a website, listservs, email, and public radio to reach residents and field workers. Public outreach enabled access to samples from private lands through the assistance and contributions of ranchers and other landowners who responded. We created a spatial–temporal RShiny tool (an open‐source web framework for creating web applications in R) at adaptable resolution for the province of BC with archived spatial data. We mapped all available Sandhill Crane breeding location information on this site, including possible, probable, and confirmed breeding location records from 1960 to 2018 from eBird (eBird 2018), the BC Breeding Bird Atlas (Davidson et al. 2015), iNaturalist (iNaturalist 2018), as well as citizen science data conveyed to us directly. The map tool guided sample collection effort by highlighting known breeding areas, and encouraged volunteers to contribute their own knowledge of previously undocumented breeding sites.

Collection of moulted feathers is a non‐invasive sampling method particularly well‐suited to cranes as their feathers are large, easily spotted, and often identifiable by staining with iron‐rich mud during the breeding season (Walkinshaw 1949). Participants were asked to collect the freshest and largest feathers, since larger wing, bustle, or tail feathers generally yield more DNA than coverts or body contour feathers (Johansson et al. 2012; Kelly 2019; Peters et al. 2020). Feathers were collected in July and August, after the nesting period, when chicks had left the nest, coinciding with the peak moulting season of 2017 and 2018 (Walkinshaw 1950). We communicated individually with volunteers to ensure feathers were collected from known breeding areas rather than staging areas of migratory cranes. Moulted feathers were retrieved within 100 m of nest sites wherever possible to prevent the collection of feathers from migrating birds. Once feathers were collected, they were dried at room temperature to limit exposure to humidity, which has been related to lower PCR performance (Vili et al. 2013). Feathers were then wrapped in plastic and shipped by mail to the University of British Columbia's Genetic Data Centre, Department of Forest and Conservation Sciences. In total just over 500 feathers were donated from three geographic regions of BC.

**FIGURE 1:**
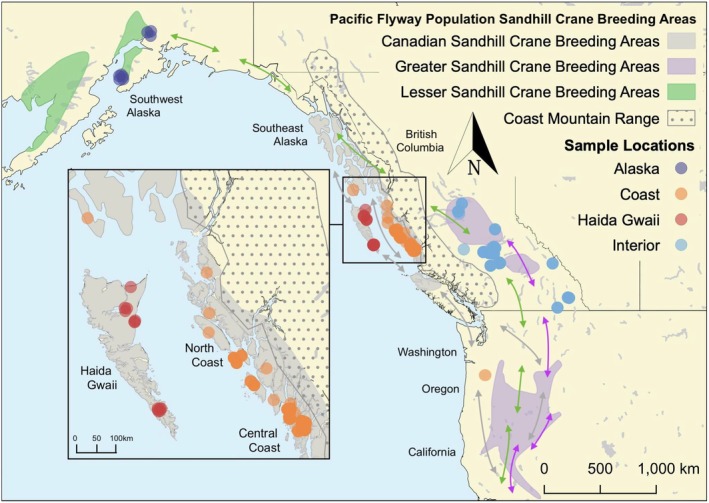
Pacific Flyway Sandhill crane breeding areas in Northwestern North America highlighting regions where the putative *A.c. canadensis* (Lesser), *A.c. rowani* (Canadian) and *A.c. tabida* (Greater) subspecies range. Coloured arrows indicate migration routes for each subspecies (Ivey 2013; Petrula and Rothe 2005). DNA sample collection sites are represented by semi‐transparent points, with colours corresponding to each of the four study regions.

Overall, the authors and our collaboration of citizen scientists collected moulted Sandhill crane feathers and other genetic material from 123 individual cranes on the Central and North Coast of BC, and from 77 cranes at various sites east of the Coast Mountains, including samples collected by 14 volunteers at 12 different Interior BC sites (Figure 1, Table 1). Additionally, we received 13 contributions of moulted feathers from Haida Gwaii collected by nine volunteers from eight separate sites. We received nine blood samples from Southwest Alaska's breeding Sandhill Cranes collected during two previous studies (Petrula and Rothe 2005; G. Ivey, unpublished data). We also received blood samples from six cranes captured and tagged while staging in the Lower Columbia River region of Washington and Oregon, and may have been part of the small (3000–5000), discrete population that winters in that region (Ivey et al. 2005, 2015; Stinson 2017). These birds were radio tagged and travelled up the coastal Pacific Flyway and summered along the BC and Southeast Alaska coast (Ivey et al. 2005).

### 2.2 Molecular Methods

We chose up to three feathers for DNA extraction if multiple feathers were collected from a single location. DNA was isolated from a total of 300 feather samples using the QIAamp DNA Investigator kit (Qiagen, Valencia, CA) following their protocol for isolation of total DNA from tissues. DNA was isolated from 15 blood samples following standard proteinase K–phenol–chloroform procedures (Sambrook et al. 1989). DNA concentration and quality was verified with a Nanodrop 2000c (Fisher Scientific, Toronto, ON, Canada). Comparison of microsatellites from each sample revealed that no duplicate microsatellite patterns were observed, suggesting that samples came from unique individuals.

**FIGURE 2:**
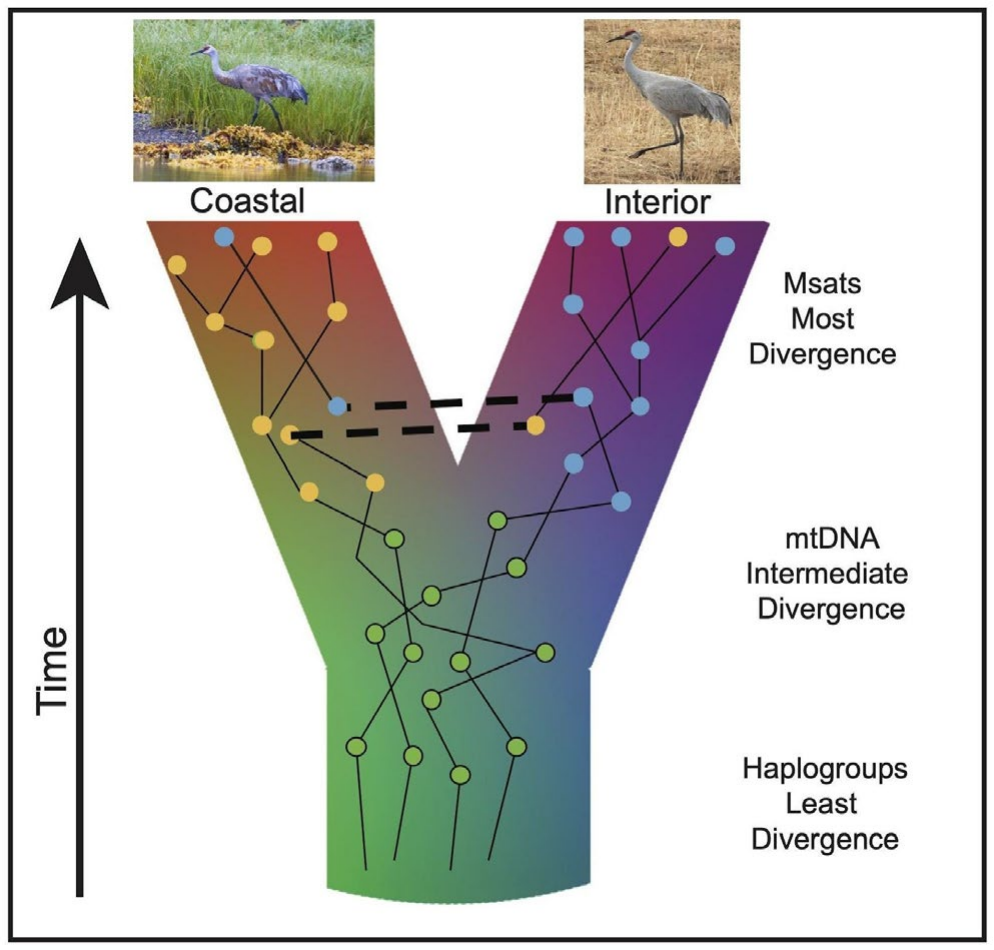
Graphical depiction of context of divergence between Sandhill Crane (*Antigone canadensis*) populations. Expected resolution of divergence of rapidly evolving microsatellite (Msats), intermediately evolving mitochondrial DNA sequences (mtDNA) and slower evolving mitochondrial haplotype groups is demarcated on the right. Between population migration (introgression) is illustrated by horizontal dashed lines.

We investigated two genetic markers to look at different resolutions of divergence times: microsatellites (Msats) and mitochondrial DNA (mtDNA) sequences. mtDNA sequences were also grouped into haplotype groups based on combinations of mutations previously shown to be informative in defining genetic variation (Hayes 2015; Figure 2). We selected these markers to capture the breadth of genetic variation in the nuclear and mitochondrial genomes, and to investigate population structure and divergence dynamics along the Pacific Flyway over different time scales. We compared differences in these markers between the four geographic regions of ‘Coast’, ‘Haida Gwaii’, ‘Interior BC’ and ‘Southwest Alaska’.

#### 2.2.1 Microsatellite Methods

We screened 28 microsatellites originally isolated from the Whooping Crane (Gram, *Grus americana* , Jones et al. 2010) and Red‐crowned Crane (Gj, *Grus japonensis* , Hasegawa et al. 1999) for cross‐species amplification and polymorphism. Of these, 8 microsatellite loci (Gram6, Gram11, Gram20, Gram22, Gram30, Gram 42 from Jones et al. (2010) and GjM15 and GjM48b from Meares et al. (2009) amplified consistently and were polymorphic). Polymerase chain reaction (PCR) was performed in 10 μL reaction volumes consisting of 2 pmol dNTP (New England Biolabs, Ipswich, MA, USA), 1X PCR buffer (Stratagene, La Jolla, CA, USA), 1.5 units of Paq5000 (Stratagene), 1 pmol each forward and reverse primer (Eurofins MWG Operon, Louisville, KY, USA), 0.3 pmol M13 IRDye labelled primer (Integrated DNA Technologies, Skokie, IL, USA), with 5–50 ng DNA template. PCR conditions followed Meares et al. (2009) and Jones et al. (2010).

Amplified products from 222 samples were denatured and run on a 5% polyacrylamide gel electrophoresed for 2.5 h on a LI‐COR 4300 automated sequencer (LICOR Inc., Lincoln, NE, USA) with a minimum of four size standards (50–350 bp or 50–700 bp LICOR) per 64 well gel and two to four reference samples. Gels were scored using SAGA 3.3 Microsatellite Analysis software (LICOR). Due to challenges associated with the extraction and amplification of DNA from feather samples, we were unable to amplify DNA from several feathers; these samples were eliminated from further analysis. To minimise potential bias in our analysis, we restricted the full dataset to samples with complete microsatellite amplification. Of the 203 samples collected from four geographic regions west of the Rocky Mountains (Table 1), 69 samples met this criterion and were included in the primary analysis. A second analysis incorporating all 203 samples was also conducted and is presented in Supporting Information.

**TABLE 1:** Sample sizes and composition across the four western crane populations. Results follow the filtered sample results that include samples with no NULL genotypes (*n* = 69 samples). These numbers and rates are reported for the full dataset in Table S1.

Sample types and descriptive statistics	Population				Overall
	Coast	Haida Gwaii	Interior BC	SW Alaska	
Number of all feathers samples	102	13	73	0	188
Number of all blood/tissue samples	6	0	0	9	15
All genotyped samples	108	13	73	9	203
Number of feathers without missing alleles	24	7	31	0	62
Number of blood samples without missing alleles	5	0	0	2	7
All samples genotyped without missing alleles	29	7	31	2	69
Samples used in mtDNA sequence and haplotype analysis	20	5	20	7	52
Samples successfully genotyped	29	7	31	2	69
Average alleles per locus	7.13	4.75	8.50	2.63	5.75
Standardised allelic richness (AR)	3.86	3.76	4.20	4.30	4.03
Mean amplification rate	1.00	1.00	1.00	1.00	1.00
Expected heterozygosity	0.62	0.68	0.58	0.69	0.64
Observed heterozygosity	0.72	0.72	0.75	0.78	0.75
Inbreeding within subpopulation (*F*_IS_)	0.19	0.07	0.24	−0.04	—
*p*‐value associated with *F*_IS_	0.001*	0.23	0.001*	0.16	—

* Significant at = 0.05 using a Benjamini‐Yekutieli correction.

To evaluate potential genotyping artefacts, we tested for linkage disequilibrium (LD) and allelic dropout among microsatellite loci. LD was assessed using the Index of Association (Ia) and the standardised measure *r̄*_*D*_ as implemented in the R package poppr v2.9.4 (Kamvar et al. 2014, 2015). Significance was evaluated with 999 random permutations of the dataset. Pairwise LD was also evaluated to identify non‐independent locus combinations. We assessed potential allelic dropout using multiple approaches. First, we tested for departures from Hardy–Weinberg equilibrium (HWE) at each locus and examined patterns of observed versus expected heterozygosity to identify excess homozygosity. Second, we implemented Brookfield's (1996) method to estimate null allele frequencies for each locus, which can be indicative of allelic dropout. These calculations were performed in R using a custom script.

#### 2.2.2 Mitochondrial Sequence Methods (mtDNA and Haplotypes)

We amplified a 437 bp portion of the mtDNA control region, using primers L‐17 and H393 (Glenn et al. 2002) for 52 crane samples, 41 feathers, and 11 blood samples (Table 1). Polymerase chain reaction (PCR) was performed in 50 l reaction volumes consisting of 0.2 mM dNTP (New England Biolabs, Ipswich, MA, USA), 1X PCR buffer (Stratagene, La Jolla, CA, USA), 2.0 units of Paq5000 (Stratagene), 50 pmol eac ofh forward and reverse primers (Integrated DNA Technologies, Skokie, IL, USA), 1.25 mg BSA, with 40 ng DNA template. PCR amplification followed Glenn et al. (2002); purification and sequencing of PCR products followed Hersh et al. (2022).

Sequence chromatograms were imported into Geneious 8.1.9 (Biomatters Inc. San Diego, CA, USA). We edited and assembled the forward and reverse sequences for each sample into individual contigs, checking that results were the same for the complementary strands. We inspected alignments by eye for ambiguous base calls, paying particular attention to confirm nucleotide polymorphisms. Consensus sequences were aligned and exported from Geneious (Geneious 8.1.9) in FASTA format for haplotype analysis and assessed with DNAdiffer (Ritland 2012) to assign haplotypes.

Fifty‐two individual mtDNA sequences, 437 bp in length, were analysed (Geneious 8.1.9, DNAdiffer 2012). These sequences represented 22 unique haplotypes, which were aligned with reference sequences from Genbank (Accession AF367871, Group A; AF367890, Group B; AF367905, Group C as described by Glenn et al. 2002). Glenn et al. (2002) used restriction enzyme digests (HaeIII) to define these groups, and we also searched our sequences for HaeIII restriction enzyme sites using Geneious version 8.1.9. We found 3 haplogroups which directly correlated to groups of Glenn et al. (2002).

### 2.3 Population Genetic Structure

For each of the four sampling regions (SW Alaska, Coast, Haida Gwaii, and Interior BC), we used GenoDive 3.05 (Meirmans 2020) to calculate microsatellite allelic richness, allelic richness standardized by sample size (AR), expected heterozygosity (He), and observed heterozygosity (Ho). We assessed each of the four populations for the degree of inbreeding by calculating the proportion of the variance in the population contained in an individual (*F*_IS_). Deviations from Hardy‐Weinberg equilibrium were evaluated for each population at each locus and across all populations at all loci.

#### 2.3.1 Analysis of Molecular Variance

We investigate population structure and genetic differentiation in microsatellites between regional population groupings using an analysis of molecular variance (AMOVA; Excoffier et al. 1992). We tested for significant differences among populations in measures of within and between genetic diversity using an AMOVA as implemented in GenoDive 3.05, following methods of Excoffier et al. (1992) and Meirmans (2012) using an infinite allele model and 1000 permutations. Species comparisons were further evaluated for significance with post hoc pairwise tests. Post hoc (*F*_ST_) comparisons of populations allowed us to evaluate pairwise population differences (Coast vs. Interior; Coast vs. SW Alaska; Interior vs. SW Alaska; Coast vs. Haida Gwaii, Interior vs. Haida Gwaii, SW Alaska vs. Haida Gwaii). Statistical significance was tested using 1000 permutations, and tested at significance level α=0.05.

#### 2.3.2 Isolation by Distance

To evaluate isolation by distance between Sandhill Crane populations, we first estimated pairwise genetic differentiation between all four sampling regions in this study using Wright's *F*‐statistic (*F*_ST_, Wright 1943), calculated using GenoDive 3.05 (Meirmans 2020). We then calculated the geographic centroid for each study population and evaluated the relationship between pairwise geographic distances and pairwise genetic distances in R (R Core Team 2023). Mantel tests were performed as implemented in the R package adegenet (Jombart 2008).

#### 2.3.3 Discriminant Analysis of Principal Components

Discriminant Analysis of Principal Components (DAPC; Jombart and Collins 2015) was used to investigate the genetic structure of biological populations. DAPC integrates the sampling region prior into a variance optimization function through the rotation of axes that maximizes the between‐region variance while minimizing the within‐region variance (Jombart 2008). We ran successive *K*‐means clustering analyses on linear discriminant functions of one, two, and four sampling clusters and used the Bayesian Information Criterion (BIC; Schwarz 1978) to assess the best supported model. We used the package adegenet that implements DAPC in R (Jombart 2008).

#### 2.3.4 Bayesian Cluster Analysis

To compare against a priori grouping of individuals in the above analyses, further examination of microsatellite genetic structure between and within crane populations was performed using a Bayesian model‐based cluster analysis implemented in Structure V2.3.4 (Pritchard et al. 2000). The methods in the Structure clustering approach are individual‐based and are aimed at reducing the amount of bias that sampling and grouping have on the determination of population structure (Falush et al. 2003), particularly in comparison to the DAPC method above. Structure implements Bayesian methods by calculating the likelihood that the data can be divided into a number of clusters, with each cluster trying to maximize Hardy–Weinberg equilibrium and linkage equilibrium (Pritchard et al. 2000; Falush et al. 2003; Hubisz et al. 2009). By optimizing the likelihood for different numbers of genetic clusters, we test for the optimal number in our samples restricted to those without null alleles (*n* = 69) and the full dataset (*n* = 203).

We performed 1000 Bayesian model‐based cluster simulations using the admixture and correlated allele frequency models for different numbers of clusters (*K*) for *K* = 1 through *K* = 8. We ranked models to determine the most likely cluster number based on the Bayesian information criterion (BIC) where smaller BIC indicated better parsimony (Falush et al. 2003; Hubisz et al. 2009). For each model, we set the burn‐in period to 100,000 and used 200,000 Markov chain Monte Carlo repetitions. We used all samples without null alleles in our dataset (*n* = 69) to ensure we had the best complement of samples across the four populations. We selected the model with the lowest BIC in ‘Structure’ to describe the population structure, but followed up with analyzing the data based on the restricted dataset that had all 8 loci amplified to reduce potential bias from null alleles. The full analysis is reported within the Supporting Information.

#### 2.3.5 Phylogenetic Inference

Phylogenetic trees were inferred for microsatellite markers (*n* = 69, and *n* = 203) and mitochondrial DNA sequences (*n* = 52). For microsatellite markers, genetic distance matrices of Sandhill Crane populations were inferred using Nei's genetic distance (*G*_ST_, Nei 1973) and Wright's *F*‐statistic (*F*_ST_) distance (Wright 1951). Nei's genetic distance and *F*_ST_ distances were calculated as implemented in PoptreeW (Takezaki et al. 2014). Phylogenetic trees were inferred from the genetic distance matrices via neighbour joining, and nodal support values were calculated based on 1000 bootstrap replicates (Takezaki et al. 2014). Resulting phylogenetic trees were visualised using FigTree (Rambaut 2012).

All obtained mitochondrial sequences (*n* = 52) were aligned using MAFFT v7.490 (Katoh and Standley 2009) and visually inspected using AliView v1.26 (Larsson 2014). A maximum likelihood phylogeny was inferred using IQTREE2 (Minh et al. 2020) following the selection of the most appropriate substitution model using model finder (Kalyaanamoorthy et al. 2017) and nodal support values were based on 1000 bootstraps.

## 3 Results

### 3.1 Specimen Collection

The full dataset included a collection of 203 genetic samples of feathers, blood, and tissue samples. As the samples collected had problems amplifying the DNA for unknown reasons (degraded feathers, insufficient DNA, problems with mutation in the primer binding site), we were concerned about incomplete microsatellites and the potential for bias in the analysis. For example, the amplification rate of blood/tissue samples (0.810, *n* = 18) was marginally better than that of feather samples (0.75, *n* = 185), but this difference in amplification was not significant (*p* = 0.13).

### 3.2 Microsatellite Genetic Diversity and Structure

Mean amplification rate varied both across sampling regions and between loci and is reported in Table S1. Mean amplification ranged from 0.682 to 0.885 among the four geographic regions (Table S1). Of the eight loci examined, the rate of amplification varied from 0.581 to 0.818 with Gram11 and GiM15 having the highest amplification rates (Table S2). The mean amplification across all loci was 74.7%. For this reason, we made the decision to subset the full dataset to only include those samples with complete genotypes (samples with no missing alleles) in the population analysis, resulting in a primary dataset composed of 69 of the 203 original samples that met this amplification criteria (Table 1).

Allelic diversity varied across populations, with Interior BC showing the highest richness (8.50 alleles/locus; *n* = 31) and Coast population lower (7.13; *n* = 29). Haida Gwaii (5.75; *n* = 7) and SW Alaska (2.63; *n* = 2) had reduced diversity, but these estimates may be biased downward by small sample sizes. The average alleles per locus and standardised allelic richness (AR) per locus were 5.75 and 4.03 for Haida Gwaii and SW Alaska within the full dataset (including incomplete genotypes (Table S1)).

We tested for linkage disequilibrium (LD) among loci using exact tests (Rousset 2008), applying Bonferroni corrections to account for multiple comparisons. We detected multilocus linkage disequilibrium across the full dataset (*r̄*_*D*_ = 0.041, *p* = 0.001). However, when analysed by population, this signal was largely restricted to the coast population (*r̄*_*D*_ = 0.052) while Haida Gwaii showed no evidence of LD (*r̄*_*D*_ = −0.029), and the interior population exhibited a weaker signal (*r̄*_*D*_ = 0.033). The SW Alaska population could not be evaluated due to insufficient genetic variation. Pairwise LD analysis identified several locus pairs with elevated *r̄*_*D*_ values, notably Gram6:Gram11, Gram42:GjM15, and Gram22:GjM48b. Null allele frequencies were mostly low, but two loci (GjM15 and GjM48b) showed slightly higher frequencies, suggesting possible allelic dropout or null alleles. To evaluate their impact, we excluded these loci and reran the analyses yielding similar results. This confirmed their removal did not significantly affect population structure or genetic differentiation patterns.

### 3.3 Population Structure

As the inbreeding coefficient, *F*_IS_, can be sensitive to allelic dropout (Soulsbury et al. 2007), we report our results on samples without allelic dropout (*n* = 69). Significant inbreeding, or deviations from Hardy Weinberg equilibrium within populations, was detected in the Coast (*F*_IS_ = 0.19, *p* = 0.001) and Interior BC (*F*_IS_ = 0.24, *p* = 0.001), indicating heterozygote deficits were unlikely due to inbreeding or undetected null alleles. The Haida Gwaii (*F*_IS_ = 0.07, *p* = 0.23) and SW Alaska (*F*_IS_ = −0.04, *p* = 0.16) populations showed no significant deviations reflecting the small sample size. The *F*_IS_ for the SW Alaska population within the full dataset of *n* = 203 indicated significant inbreeding (or undetected null alleles), but remained non‐significant for the Haida Gwaii population (Table S1).

**FIGURE 3:**
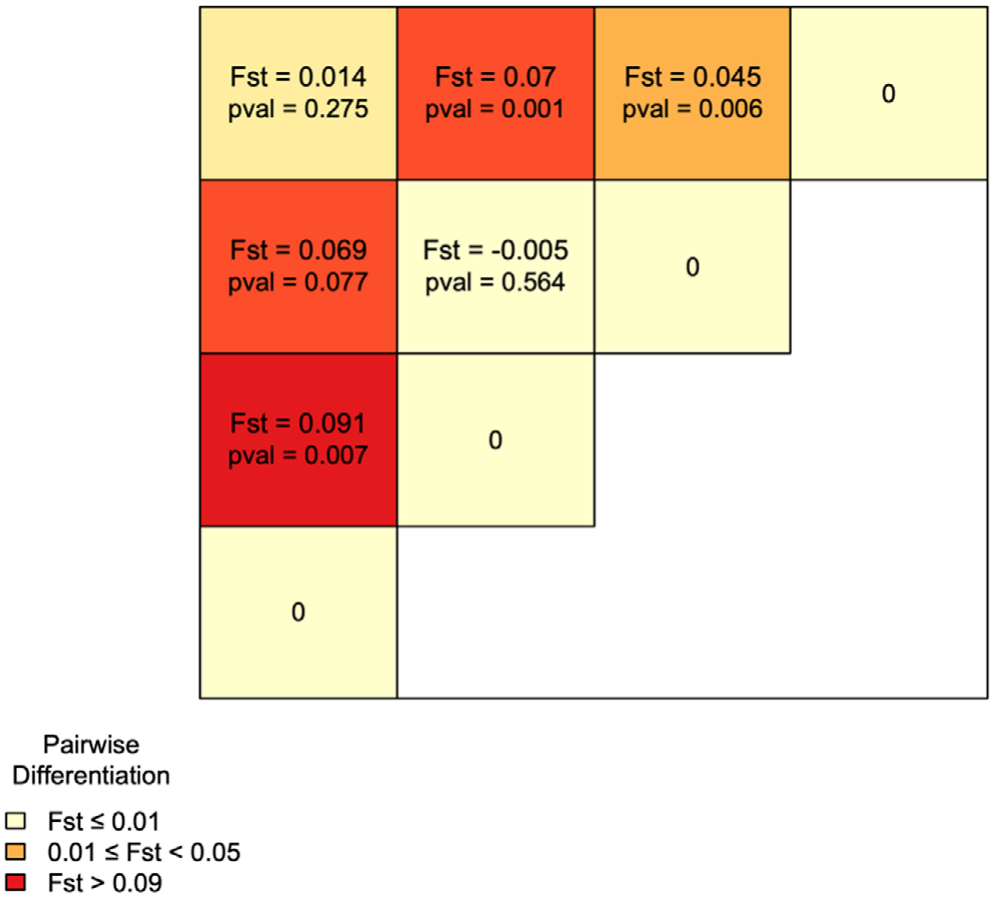
Values of *F*_ST_ for pairwise differentiation of populations where darker colours represent populations with greater genetic divergence. *p*‐values below the significance threshold indicate evidence of differentiation from HWE. *F*_ST_ values between 0.05 and 0.25 indicate moderate genetic differentiation (Wright 1978).

Degree of differentiation between populations as measured by *F*_ST_ indicates that 5% (*p* = 0.001) of the total genetic variability can be attributed to differences among populations. The overall AMOVA results suggest significant differences between sampling regions (*p* = 0.001). Post hoc, pairwise analyses for differences between sampling regions suggested that Haida Gwaii and Coastal BC samples were significantly different from samples from Alaska and Interior BC (*p* < 0.05, Figure 3). By contrast, birds from Interior BC/Alaska sampling regions (*p* = 0.275), and Haida Gwaii/Coastal BC sampling regions were not statistically different (*p* = 0.564; Figure 3).

Private alleles occurred in 6 of 8 loci within *n* = 69 samples (Table 2). Within the Coast population, 13% of alleles (11 of 83) were private, and the Interior BC population had 24% (20 of 83 alleles). Sample sizes were too small to report private allele rates in SW Alaska and Haida Gwaii. When the most similar populations were grouped (Alaska with Interior, Coastal with Haida Gwaii) six of the loci contained private alleles in each of the Alaska/Interior and the Coast/Haida Gwaii populations (Table 2). The majority of alleles were shared between both grouped populations (44 of 83). However, 18% of alleles were private (unique) to Coastal cranes (Coast and Haida Gwaii cranes) and 27% were unique to the SW Alaska and Interior BC cranes (Table 2). These percentages from the constrained dataset (*n* = 69) are similar to those reported for the full dataset (*n* = 203) (Table S3). We assessed the rate of null alleles for all eight loci and found two loci exceeding the threshold of 0.10 (Table 2). However, we retained these loci in our analyses because they remained below the more conservative threshold of 0.20 recommended by Chapuis and Estoup (2007) and Dakin and Avise (2004) when evaluated on the full dataset (*n* = 203; Table S3).

**TABLE 2:** Number of shared and private alleles across four populations: Alaska, Interior BC, Coastal BC, and Haida Gwaii as well as those shared between populations grouped based on similarity (Alaska/Interior and Coast/Haida Gwaii). The significance of the test for Hardy Weinberg Equilibrium (HWE) for all 8 loci is shown; the overall *p*‐value of all samples was < 0.001.

Locus name	Gram‐6	Gram‐11	Gram‐20	Gram‐22	Gram‐30	Gram‐42	GjM‐15	GjM‐48b
Number of alleles	10	17	20	5	15	4	4	8
Shared alleles across four populations (%)	10.0	5.9	15.0	40.0	13.3	25.0	50.0	25.0
Shared across interior/Alaska (%)	20.0	29.4	40.0	0.0	13.3	0.0	50.0	37.5
Shared across coast/HG (%)	30.0	17.6	25.0	20.0	13.3	0.0	50.0	12.5
Private alleles in coast (%)	30.0	11.8	20.0	0	13.3	0	0	0
Private alleles in Haida Gwaii (%)	0	0	5.0	0	0	0	0	0
Private alleles in interior (%)	10.0	29.4	40.0	0	13.3	0	50.0	25.0
Private alleles in SW Alaska (%)	0	0	0	0	0	0	0	0
Null allele rate	0.02	0.03	0.12	0.01	0.0	0.10	0.21	0.22
Test for HWE *p*‐values*	0.007	0.005	< 0.001	0.028	0.003	< 0.001	< 0.001	< 0.001

* *p*‐values are corrected using the Benjamini and Yekutieli (2001) correction.

There were no significant differences (*p* > 0.05) between cranes sampled in SW Alaska vs. the Interior populations nor between the Coastal BC and Haida Gwaii populations (Figure 3). Moderate significant differences were found between the Interior and Haida Gwaii populations (*p* ≤ 0.05) and between the Coast and SW Alaska populations (*p* ≤ 0.05). Highly significant differences (*p* ≤ 0.001) were found between the Interior and Coast populations, reflecting greater statistical power. There was limited evidence of genetic differentiation between the Alaska and Haida Gwaii populations (*F*_ST_ = 0.069, *p* = 0.077) and significant differentiation between the Alaska and Coastal BC populations (*F*_ST_ = 0.091, *p* = 0.007) (Figure 3). However, these results should be interpreted cautiously due to the small SW Alaska sample size (*n* = 2) in the constrained dataset (*n* = 69). In the full dataset (*n* = 203), we found similar magnitude *F*_ST_ between SW Alaska and Coast and SW Alaska and Haida Gwaii, as well as stronger evidence of differentiation (*p* = 0.004, and *p* = 0.002 respectively) (Figure S1).

#### 3.3.1 Discriminant Analysis of Principal Components

Overall, the discriminant analysis of principal components (DAPC) model assigned individuals to correct sampling regions (66 of 69 samples) in four populations (Table 3). No mistakes were reported within the Interior BC and SW Alaska samples. The DAPC assigned one Coast sample to the Interior, another to Haida Gwaii, and assigned one Haida Gwaii to the Coast.

**TABLE 3:** Comparison of proportion of DAPC assigned to correct group (i.e., probability of a sample being correctly identified to its geographic sampling region), compared to the Structure cluster analysis' assignment to Cluster 1 and Cluster 2.

Sample population	DAPC	Structure results		
	Rate DAPC sample was correctly assigned to geographic sampling region	Modelled proportion of samples in Coast/Haida Gwaii clusters	Modelled proportion of samples in Interior/Alaska clusters	Structure clusters assigned to correct geographic sampling region
Coast	0.93	0.86	0.14	0.83%
Haida Gwaii	0.86	0.71	0.29	
Interior BC	1.00	0.10	0.90	0.91%
Alaska	1.00	0.00	1.00	

We evaluated evidence for different numbers of populations (‘1’: All sampling regions, ‘2’: Coast/HG vs. SW Alaska/Interior; and ‘4’ populations corresponding to four sampling regions; and ‘8’ subpopulations) and found the most support for the model with two populations (smallest BIC statistic: 1986, compared to 1 population (BIC: 1029.0) or 4 populations (BIC: 1029.3)).

#### 3.3.2 Bayesian Cluster Analysis

**FIGURE 4:**
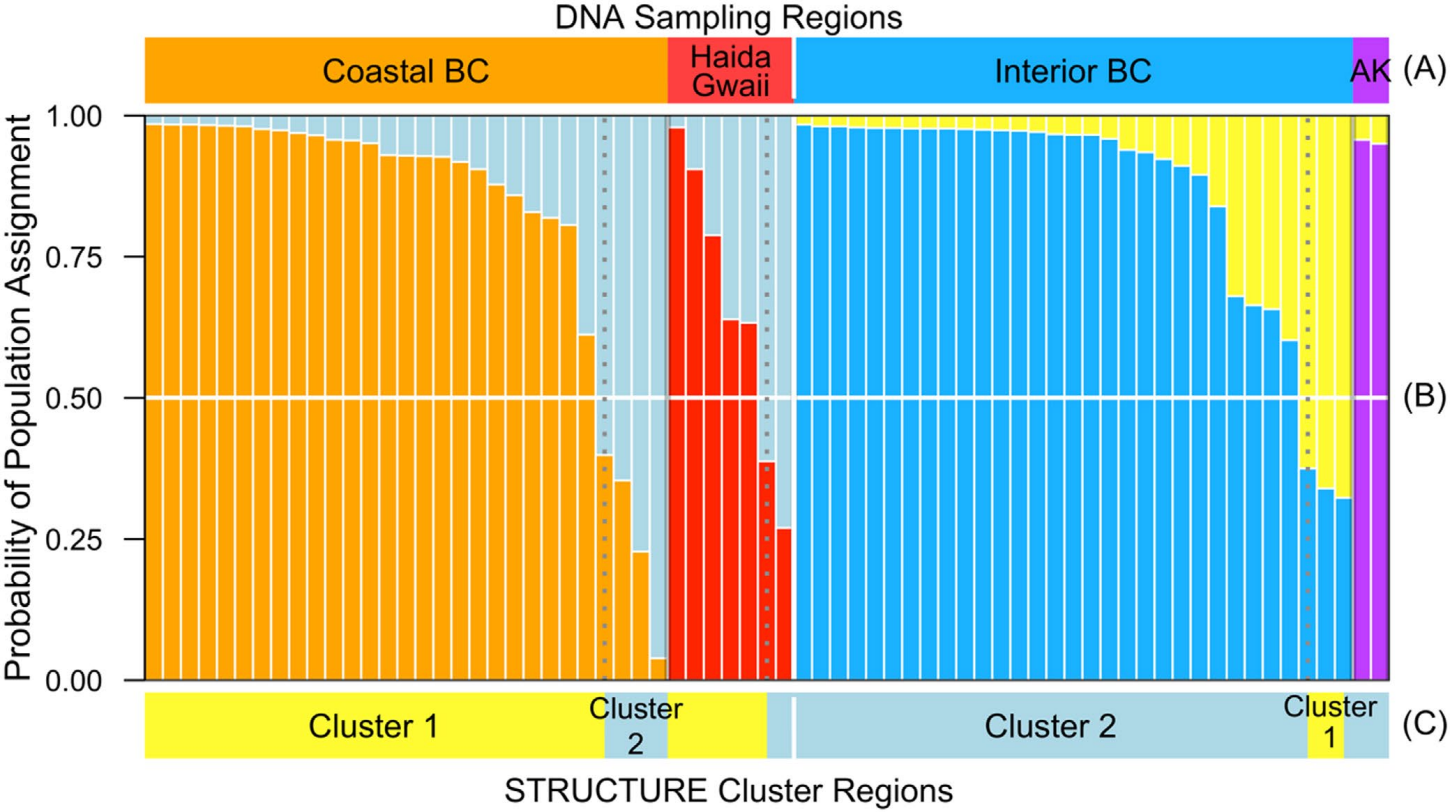
Bayesian cluster analysis of the microsatellite DNA genetic structure extracted from Sandhill Crane samples collected in four different DNA sampling regions (A; British Columbia = BC; Alaska = AK). Individual admixture proportion of ancestry assigned to individuals in each of the *K* = 2 subgroups is plotted for each individual (B). Individuals are ordered by DNA sampling region, and then by model‐based likelihood (admixture proportion) for Cluster 1 for the Coastal BC (orange) and Haida Gwaii (red) sampling regions. For Interior BC (blue) and Alaska (purple) sampling regions, individuals are ordered by the likelihood for Cluster 2. (C) If an individual has a higher admixture proportion of belonging to Cluster 1 relative to Cluster 2 it is coloured yellow, if an individual has a higher admixture proportion for Cluster 2, it is coloured light blue.

Using the Bayesian analysis software ‘Structure’, we tested evidence for the number of clusters in the genetic samples for each of *K* = 1 to *K* = 8, using the full dataset (*n* = 203). The best model with the lowest BIC was the model that returned two population clusters (*K* = 2; BIC: 1370.6 relative to the next closest model of *K* = 3, BIC: 1374.4). We assessed the population structure using the restricted dataset of *n* = 69, and *n* = 203 (Supporting Information). The majority of assignments aligned with geographic DNA sampling regions (Table 3; Figure 4) however, some admixture among populations is occurring. Cluster 1 contains the majority of Coast and Haida Gwaii samples (83.3%, Table 3), whereas Cluster 2 contains the majority of Interior BC and Alaska samples (90.6%). Broken down by sampling regions, we found 86% of Coastal BC and 71% of Haida Gwaii birds were grouped in Cluster 1, while Cluster 2 contained 90% and 100% (but *n* = 2) of the Interior BC and Alaska samples, respectively (Table 3; Figure 4).

### 3.4 Mitochondrial Genetic Diversity and Structure

**FIGURE 5:**
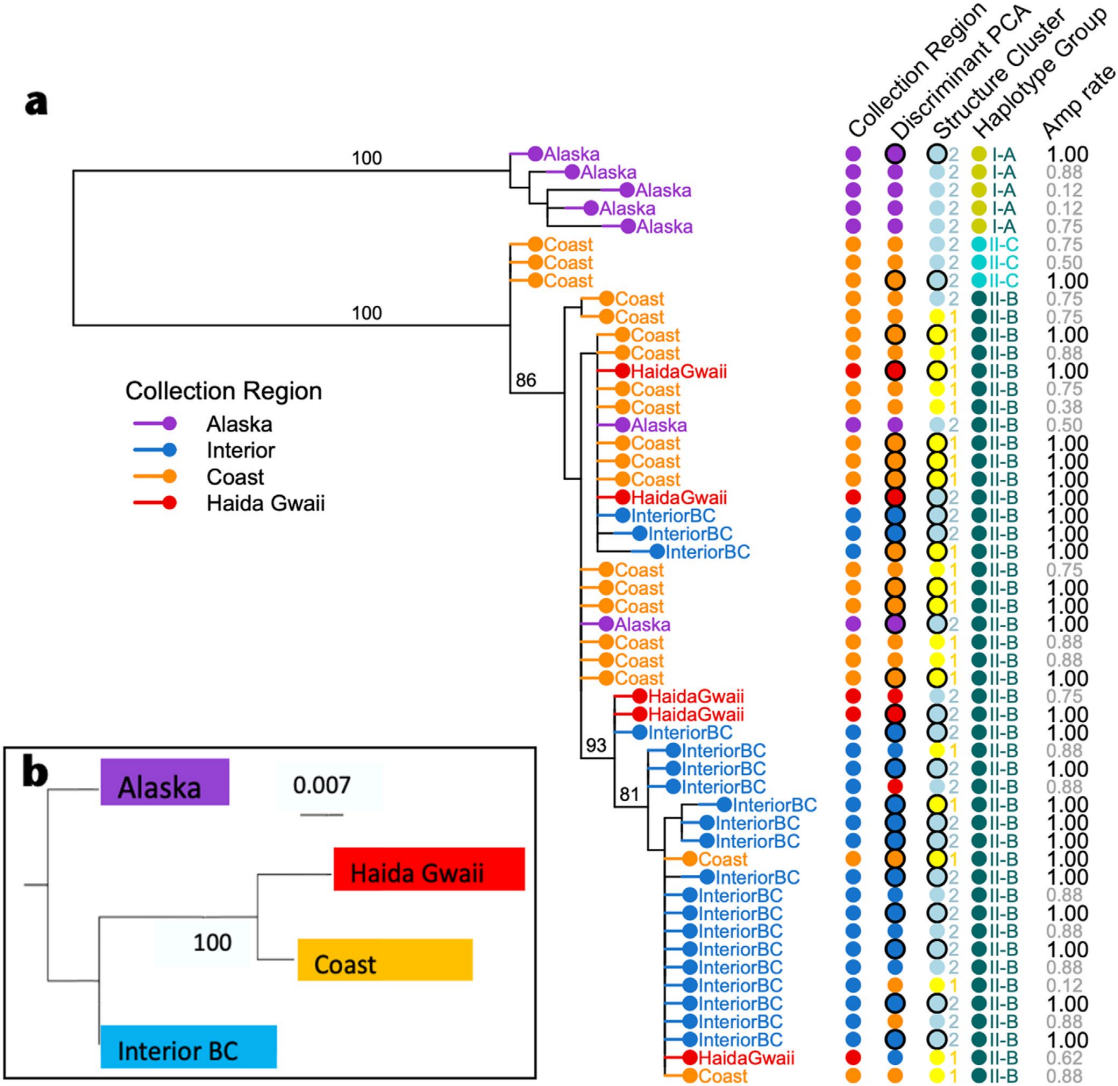
Phylogenetic relationships among Pacific flyway Sandhill Cranes. Maximum likelihood phylogenetic tree of mtDNA inferred using IQTREE2, bootstrap support values are denoted at the nodes (if support > 80) (a). Each point represents an individual crane. The black outlines around points in the DPCA and Structure Cluster columns indicates samples with amplification rates of 1.0 (‘Amp rates’). Neighbour joining phylogenetic tree inferred from *F*_ST_ distances derived from microsatellite markers in PoptreeW (b). Nodal support value is based on 1000 bootstrap replicates. Each tip represents a population. Scale bar represents *F*_ST_ genetic distance.

Sequences obtained for 52 individuals fell into 22 different haplotypes (Genbank accession numbers xxxxx‐xxxxx; Figure 5a). The three most frequent haplotypes were a, c, and k. Haplotype ‘a’ was shared by nine individuals (17% frequency): six from the Coast, two from Haida Gwaii, and one from the Interior population. Seven Coast cranes (13%) shared haplotype ‘c’ while haplotype ‘k’ was shared by seven other birds (13%); five Interior, one Haida Gwaii, and one Coast. Eleven haplotypes were unique (21%). Following Glenn et al. (2002) and Rhymer et al. (2001) reference groups, we identified three haplotype groups in our Sandhill Crane mitochondrial control region sequences from the four regions. These groups broadly correspond to clades in the phylogenetic tree. One clade consists of the highly diverged Alaska population (Group I‐A), the second, coastal clade, at the base of the remaining samples in the phylogenetic tree (Group II‐B), and the third (Group II‐C) includes all other samples from the Coast, Haida Gwaii, Interior, and Alaska groups (Figure 5a).

### 3.5 Overall Population Structure

#### 3.5.1 Phylogenetic Inference

Inferred phylogenetic relationships among sampled Sandhill crane populations based on the mtDNA control region sequences (*n* = 52) suggest there is population structure in the Pacific Flyway Sandhill Cranes (Figure 5a). The analysis primarily splits individuals into clades based on breeding region of origin with limited admixture. Bootstrap support values reveal 100% nodal support for separation between the Alaska group and all populations of BC cranes, i.e., samples collected on Haida Gwaii, the Coast, and in Interior BC. There is good nodal support (93%) for the clade, that is composed primarily of samples derived from the BC Interior breeding population (blue samples in the lower clade of Figure 5b). Coast and Haida Gwaii populations are largely separate from Alaska and Interior clades but without strong nodal support and with some introgression or incomplete lineage sorting.

An additional phylogenetic tree based on microsatellite markers inferred using F_ST_ distances (Figure 5b) is generally in broad agreement with the mtDNA phylogeny. However, the microsatellite phylogenies reveal strong support (nodal support of 100%) for the node separating the Coastal BC and Haida Gwaii birds from the Interior BC and Alaska birds, indicating support for the split between the Coastal and Haida Gwaii populations relative to the Interior BC and Alaska populations. There was no difference in topology for trees inferred under *F*_ST_ distances and those inferred using Nei's genetic distance (*G*_ST_; Nei 1973).

#### 3.5.2 Isolation by Distance

**FIGURE 6:**
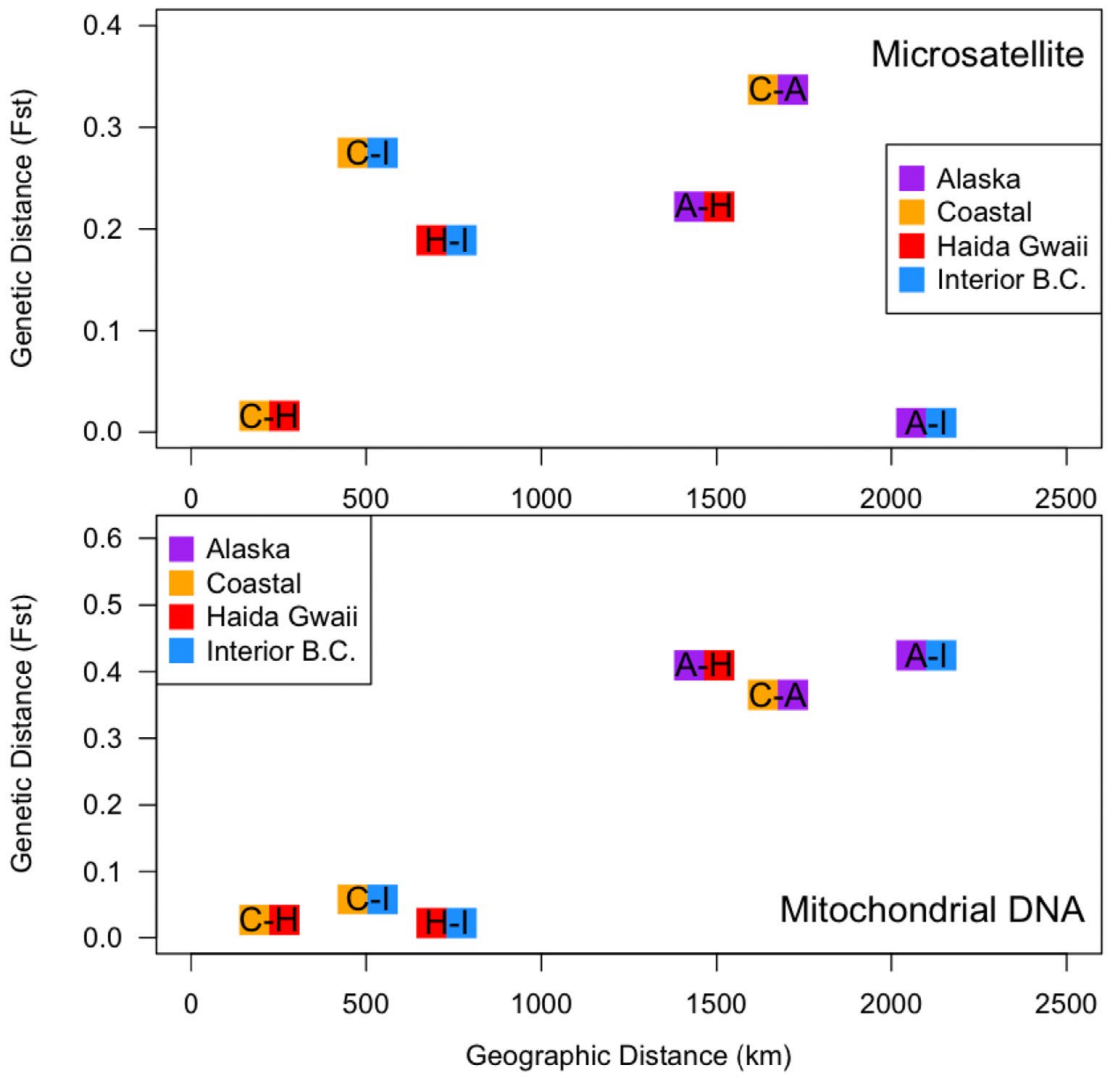
Correlation between genetic distance (as judged by *F*_ST_) and geographic distance. The top panel shows this relationship for microsatellite data, whereas the bottom panel shows the relationship for mtDNA. In both cases, there is evidence of large genetic distances and large geographic distances between all populations and Alaska.

We estimated pairwise genetic differentiation for microsatellite and mitochondrial DNA between the four sampling regions in this study. Differentiation of microsatellite *F*_ST_ followed the expected differentiation with geographic distance. The Interior BC and Alaskan populations were the farthest geographic distance but suggested the closest genetic distance (Figure 6, top panel; Figure S3 for *n* = 203 analysis). This pattern was not repeated in pairwise differentiation of mtDNA, where the four populations displayed a pattern consistent with isolation by distance (Figure 6, bottom panel) in which the strong effect of large genetic distances and large geographic distances between all populations and Alaska was observed.

## 4 Discussion

Our integrated analyses across a range of genetic markers reveal differences in the degree of genetic differentiation between geographically separated breeding populations of Sandhill Cranes in the Pacific Flyway. These results support the results of previous studies analysing morphological, habitat, and genetic data. Previous genetic and morphological studies have identified migratory cranes in regions east of the Rocky Mountains as belonging to two distinct subspecies, *A.c. tabida* and *A.c. canadensis*, with samples from *A.c. rowani* either grouping together with those from *A.c. tabida* (Rhymer et al. 2001; Glenn et al. 2002) or being intermediate (Petersen et al. 2003; Jones et al. 2005). Ivey et al. (2005) found that cranes wintering in Sauvie Island and Ridgefield National Wildlife Refuge (Lower Columbia River, Washington State) migrated west of the coast mountains to summer breeding grounds ranging from the central coast of BC to southeast Alaska (Dall Island and Prince of Wales Island). The morphological data and general features (large flat head profiles on intermediate body size; shorter unfeathered portion of legs) suggest that these birds are from the *A.c. rowani* subspecies. A genetic analysis using amplified fragment length polymorphism (AFLP) of eight coastal breeding cranes found they grouped together and were separate from Alaskan cranes, but with incomplete separation from interior (Oregon and California) nesting cranes (Hayes 2015) although no Interior BC cranes were included in that study.

Analysis of the assignment of mitochondrial control region sequences into haplogroups reveals variation between the southwest Alaska population of *A.c. canadensis* and all regions sampled in British Columbia and southeast Alaska. Phylogenetic trees of mtDNA and microsatellites illustrate population structure emerging among geographically isolated coastal and interior breeding populations with limited introgression or incomplete lineage sorting. Overall, more rapidly evolving genetic markers (microsatellites) show higher levels of genetic differentiation among geographically isolated breeding populations compared to intermediate mtDNA sequence and more conserved mtDNA haplogroups that show the least differentiation.

Multiple lines of evidence suggest coastal populations of Sandhill Cranes are genetically differentiated from the *A.c. tabida* of interior BC, and the *A.c. canadensis* found in southwest Alaska. The results of AMOVA, DAPC, and Structure k‐means clustering algorithms of microsatellite loci all suggest significant differentiation between Coastal BC regions and the Interior BC and Alaska regions. Phylogenetic trees based on both microsatellite and mtDNA sequence data displayed broad agreement in the separation of major clades by geographic region, with mtDNA showing limited introgression and weak nodal support values. Microsatellite phylogenies display strong support for the node separating coastal cranes from Interior BC and Alaska birds, indicating support for the split between the Coast and Haida Gwaii population relative to the Interior BC and Alaska populations. Finally, genetic distance (*F*_ST_, *G*_ST_ of microsatellites) was correlated with genetic distance between Coastal and Interior populations but did not correlate with geographic distance between Sandhill Crane populations of the coastal region of Southwest Alaska and Interior BC. Overall, these analyses of available genetic markers highlight population structure within the Pacific Flyway population that broadly corresponds with ecological divisions, and the importance of considering the distinctive ecological context of Coastal and Interior Sandhill Crane populations in conservation and management planning.

Genetic divergence between Coastal, Interior, and Southwestern Alaskan cranes could be due to historical or contemporary factors or a combination. Jones et al. (2005) postulated that a glacial barrier during the Pleistocene era caused a geographic constraint that may have been responsible for the initial separation of the northern *A.c. canadensis* and southern *A.c. tabida* subspecies; however, the separation time was not long enough for full reproductive isolation and speciation. This breeding isolation may have been long enough to give rise to the haplotype variation between Sandhill Cranes subspecies west of the Rocky Mountains. If the vicariant glacial barrier of this era was responsible for the formation of these two subspecies, then its subsequent melting, beginning 18,000 years ago at the western margin (Darvill et al. 2018), might have resulted in these two subspecies coalescing, depending on the degree of gene flow between populations. Haplotype group I‐A, typically associated with *A.c. canadensis* (Glenn et al. 2002; Rhymer et al. 2001) was only detected in the Alaskan Bristol Bay and Cook Inlet breeding birds, whereas in our study there were two cranes with haplotype group II, typically associated with *A.c. rowani* and *A.c. tabida*, found in southwest Alaska. Both cranes were male radio‐tagged birds, suggesting there may be male‐mediated dispersal from the southern populations up into Alaska.

It is possible that Coastal and Haida Gwaii cranes persisted through the Last Glacial Maximum of the Pleistocene in glacial refugia on the outer coasts of southeast Alaska and BC's northern coast or recolonized these areas early afterwards. Recent studies of pollen in glacial sediments suggest that the Hecate Plain, now submerged under Hecate Strait, between Haida Gwaii and the North and Central Coast, provided a glacial refugium that may have supported a diverse flora and fauna during the Fraser glaciation (Mathewes and Clague 2017). Part of the coastal crane habitat, the island archipelago of Haida Gwaii, may have been ice‐free during the last ice age. The existence of Haida Gwaii as a glacial refugium has been suggested by the presence of multiple endemic taxa (Foster 1963; Heusser 1990) and possibly unique genetic lineages (O'Reilly et al. 1993; Soltis et al. 1997; Byun et al. 1997; Clarke et al. 2001; Smith et al. 2001; Fleming and Cook 2002; Topp and Winker 2008). For example, divergence estimates for endemic subspecies of the sedentary Northern Saw‐whet Owl, Hairy Woodpecker, and Steller's Jay provide evidence that these likely survived throughout the last glacial maximum in the area of Haida Gwaii (Pruett et al. 2013). Evidence of a forested glacial refuge along the western edge of the Alexander Archipelago in Southeast Alaska was also suggested by genomically distinct clades within Pacific martens ( *Martes caurina* ) on the North Pacific Coast (Colella et al. 2019). Pollen records show the development of pine ( *Pinus contorta* subsp. *contorta*) parkland vegetation in the Western Alexander Archipelago of southeast Alaska soon after the LGM (∼15,240–14,040 ybp), suggesting recolonization from coastal refugia (Ager 2019).

Another possibility that could explain historical genetic differentiation of coastal cranes is the early recolonization of coastal areas that maintained unusually stable sea levels over the past 15,000 years. The Dundas Island Archipelago, northeast of Haida Gwaii (McLaren 2008), as well as the Hakai Passage area of the Central Coast (McLaren et al. 2014) were identified through core sampling as places with stable shorelines over this period, occupying a sea level hinge, with land depressed underwater by glaciers to the east and uplifted above sea level to the west by tectonic forces (Clague et al. 1983). Both of these areas are currently important breeding habitats for coastal cranes (Roessingh 2012).

Continued contemporary divergence could be driven by different habitat selection, foraging strategies, and behaviors for predator avoidance of Coastal and Haida Gwaii cranes compared to Interior cranes. Although Sandhill cranes are omnivorous and occupy a wide range of habitats across North America, cranes breeding in coastal BC and Haida Gwaii nest and roost in bog complexes and feed on marine mollusks within estuaries and on coastal beaches (Hearne and Hamel 2003; Roessingh 2012). Microsatellite data suggest that coastal breeding Sandhill Cranes are continuing to diverge from interior birds despite some introgression. Ecological selection can cause rapid divergence in candidate genes depending on how it affects survival and reproductive rates, but neutral markers generally show slower rates of genetic divergence relative to markers under selection. Some amount of reproductive isolation may result in genetic distinction despite low levels of gene flow when combined with sufficiently strong or divergent ecological selection. Adaptation to different breeding environments can lead to increasing reproductive isolation despite sympatry during non‐breeding periods, even without extended isolation of allopatric breeding populations (Winker 2010). Ecological traits, such as habitat associations, may correlate with patterns of genetic diversity and divergence, as shown in the comparison of closely related upland and floodplain species of Amazonian birds (Harvey et al. 2017). In cranes, such divergent ecologies may drive local adaptation and/or reproductive isolation leading to genetic differences in alleles between coastal and interior cranes over time.

Although samples collected and included in this study covered a wide geographic region in British Columbia, to gain an understanding of the whole Pacific population, samples of feather or blood from the neighbouring Pacific US states of Alaska, Washington, Oregon, and California would strengthen and build a complete subspecies level inference, putting into context the population structure observed in this British Columbia‐focused study. Furthermore, whereas all analysed datasets provided sufficient resolution to detect patterns of genetic structure, the inclusion of additional or higher‐quality microsatellite loci, as well as nuclear or mitochondrial markers, could further improve resolution and reduce uncertainty. Future work incorporating broader genomic data may help disentangle subtle population structure or confirm patterns observed in this study.

However, overall, the significant between‐population genetic differentiation observed in this study coupled with differences in migration route, diet, and breeding habitat distinguishes Coastal cranes from Interior and Alaskan populations, suggesting that Coastal cranes are on an independent evolutionary trajectory and may meet the Committee on the Status of Endangered Wildlife in Canada's criteria for a Designatable Unit (COSEWIC 2020). Overwintering habitat for western cranes in Washington, Oregon, and California is protected, but critical crane breeding habitat in BC currently has no protection. Cranes are a migratory species with summer and winter habitat separated by the Canada‐US international border, complicating conservation efforts. In BC, proposed protection of Sandhill Cranes continues to be based on a single‐species approach rather than at the ecosystem level that integrates genetic structure into how conservation of cranes and their habitat is managed. With the climate shifting across British Columbia to warmer, drier, and more fire‐prone summers, we anticipate the native range of Sandhill Cranes and other species dependent on marshlands for breeding to become a transient and dynamic property. In the coming decades, ensuring the fullest range of adaptations within local populations will ensure the resiliency of the species and will be an important prerequisite to effective stewardship (Suding and Hobbs 2009).

## Author Contributions

**Ruth Joy:** conceptualization (equal), data curation (equal), formal analysis (equal), funding acquisition (equal), methodology (equal), project administration (equal), supervision (equal), visualization (equal), writing – original draft (equal), writing – review and editing (equal). **Krista Roessingh:** conceptualization (equal), data curation (equal), funding acquisition (equal), investigation (equal), project administration (equal), resources (equal), supervision (equal), visualization (equal), writing – original draft (equal), writing – review and editing (equal). **Kathleen Meszaros:** data curation (equal), formal analysis (equal), investigation (equal), writing – original draft (equal), writing – review and editing (equal). **Allyson Miscampbell:** data curation (equal), formal analysis (equal), methodology (equal), resources (equal), writing – original draft (equal), writing – review and editing (equal). **Carol Ritland:** methodology (equal), project administration (equal), resources (equal), supervision (equal), writing – original draft (equal), writing – review and editing (equal). **Matt Hayes:** data curation (equal), investigation (equal), methodology (equal), validation (equal), writing – original draft (equal), writing – review and editing (equal). **Gary Ivey:** data curation (equal), writing – review and editing (equal). **Mike Petrula:** data curation (equal), writing – review and editing (equal). **Jeffrey B. Joy:** formal analysis (equal), methodology (equal), supervision (equal), visualization (equal), writing – review and editing (equal).

## Disclosure

Benefits generated from this research arise from the sharing of our data and results on public databases, as described above. The results of this study will help motivate conservation and management policy along the Pacific Flyway for Sandhill Cranes.

## Conflicts of Interest

The authors declare no conflicts of interest.

## Supporting information


Appendix S1.

## Data Availability

All mitochondrial DNA sequences are deposited in GenBank under the accession numbers XXX. All metadata associated with the collection of feather and blood samples collected and used in the microsatellite, mitochondrial sequence, and haplotype analyses are uploaded as online supplemental material (Appendix S1). The microsatellite dataset used in our analyses is deposited in the Dryad database at: https://doi.org/10.5061/dryad.wwpzgmsw6.
