# Supplementary material for: Deciphering Cryptic Population Structure in Western Sandhill Crane Subspecies (Antigone canadensis) of the Pacific Flyway
Source: Ecol Evol. 2025 May 28;15(6):e71475. doi: 10.1002/ece3.71475 (PMC12119177; doi:10.1002/ece3.71475)
Supplement: Supplementary file 1 — Appendix S1. [file ECE3-15-e71475-s001.docx]

SUPPLEMENTARY MATERIAL

Supplementary Table 1: Summary of all feather and blood samples including samples containing loci with <1.0 amplification rate (n=203, includes partial genotypes). Descriptive statistics for each population derived from multiloci microsatellite DNA genotypes.

| **Sample Types and Descriptive Statistics** | **Population** | | | | **Overall** |
| --- | --- | --- | --- | --- | --- |
|  | Coast | Haida Gwaii | Interior BC | Alaska |  |
| Number of feathers samples | 102 | 13 | 73 | 0 | 188 |
| Number of all blood samples | 6 | 0 | 0 | 9 | 15 |
| All genotyped samples | 108 | 13 | 73 | 9 | 203 |
| Average alleles per locus | 9.375 | 5.500 | 9.125 | 5.375 | 7.34 |
| Standardized allelic richness (AR) | 3.920 | 3.630 | 4.200 | 4.340 | 4.02 |
| Mean amplification rate | 0.601 | 0.885 | 0.779 | 0.736 | 0.747 |
| Expected heterozygosity | 0.725 | 0.688 | 0.748 | 0.754 | 0.730 |
| Observed heterozygosity | 0.584 | 0.678 | 0.547 | 0.642 | - |
| Inbreeding within subpopulation (F_IS_) | 0.194 | 0.015 | 0.269 | 0.149 | - |
| p-value associated with F_IS_ | **0.001 | 0.395 | **0.001 | *0.022 | - |

**significant at=0.05 using a Benjamini-Yekutieli correction

Supplemental Table 2. Rate of genotype amplification and rate of null alleles by loci for the blood/tissue samples, feathers, and for all samples (combined amplification rate) (n=203).

| Amplification rate | Gram6 | Gram11 | Gram20 | Gram22 | Gram30 | Gram42 | GjM15 | GjM48b | Overall |
| --- | --- | --- | --- | --- | --- | --- | --- | --- | --- |
| Blood/Tissue (n=18) | 0.78 | 0.83 | 0.72 | 0.89 | 0.89 | 0.67 | 0.83 | 0.83 | 0.81 |
| Feathers (n=185) | 0.78 | 0.82 | 0.72 | 0.76 | 0.78 | 0.75 | 0.82 | 0.57 | 0.75 |
| All (Blood & Feathers, n=203) | 0.78 | 0.82 | 0.72 | 0.77 | 0.79 | 0.74 | 0.82 | 0.58 | 0.75 |
| Null Allele Rate | 0.04 | 0.07 | 0.14 | 0.01 | 0.04 | 0.14 | 0.18 | 0.19 | 0.10 |

**Supplemental Table 3**. Number of shared and private alleles across four populations: Alaska, Interior BC, Coastal BC and Haida Gwaii as well as those shared between populations grouped based on similarity (Alaska/Interior and Coast/Haida Gwaii). The rate of amplification at each of the 8 loci is reported with a mean amplification rate across all alleles of 0.75. The significance of the test for Hardy Weinberg Equilibrium (HWE) for all 8 loci is shown.

| Locus Name | Gram-  6 | Gram-11 | Gram-20 | Gram-22 | Gram-30 | Gram-42 | GjM-15 | GjM-48b |
| --- | --- | --- | --- | --- | --- | --- | --- | --- |
| Number of Alleles | 12 | 17 | 22 | 6 | 21 | 6 | 4 | 8 |
| Shared Alleles Across Four Populations (%) | 8.3 | 23.5 | 18.1 | 50 | 23.8 | 33.3 | 50 | 25 |
| Private Alleles in Alaska (%) | 16.7 | 0 | 0 | 0 | 0 | 0 | 0 | 0 |
| Private Alleles in Interior (%) | 0 | 5.9 | 31.8 | 0 | 4.7 | 0 | 0 | 12.5 |
| Allele in grouped Alaska/Interior Population (%) | 0 | 5.9 | 0 | 16.7 | 0 | 0 | 0 | 0 |
| Private Alleles in Coast (%) | 8.3 | 5.9 | 13.6 | 0 | 23.8 | 33.3 | 0 | 0 |
| Private Alleles in Haida Gwaii (%) | 0 | - | 4.5 | 0 | 0 | 0 | 0 | 0 |
| Private Allele in grouped Coast/Haida Gwaii Population (%) | 8.3 | - | 4.5 | 0 | 9.5 | 0 | 0 | 0 |

| Test for HWE p-values** | <0.001 | <0.001 | <0.001 | 0.019 | <0.001 | <0.001 | <0.001 | <0.001 |
| --- | --- | --- | --- | --- | --- | --- | --- | --- |

** p-values are corrected using the Benjamini and Yekutieli (2001) correction

**Supplemental Table 4**. Comparison of proportion of DAPC assigned to correct group (i.e., probability of a sample being correctly identified to its geographic sampling region), compared to the Structure cluster analysis’ assignment to Cluster 1 and Cluster 2 (n=203, includes partial genotypes).

| Sample Population | DAPC | Structure |
| --- | --- | --- |
|  | Rate DAPC sample was correctly assigned to geographic sampling region | Structure clusters assigned to geographic sampling region |
| Coast | 0.97 | 0.97 |
| Haida Gwaii | 1.0 |  |
| Interior BC | 0.84 | 0.85 |
| Alaska | 0.89 |  |


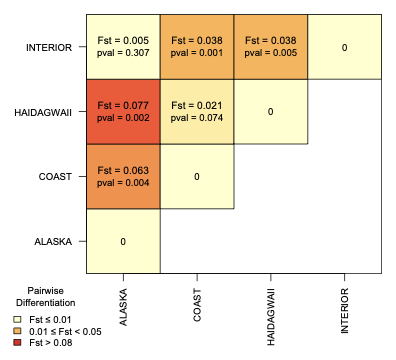


**Supplemental Figure 1**. Values of F_ST_ for pairwise differentiation of populations for the full dataset of 203 samples (including samples with null analyses). Darker colours indicate greater genetic divergence. p-values for each comparison are denoted below the F_ST_ value for each population pair (n=203, includes partial genotypes).


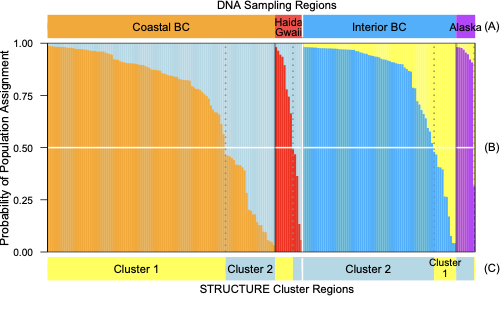


**Supplementary Figure 2.** Bayesian cluster analysis of the microsatellite DNA genetic structure extracted from Sandhill Crane samples collected in four different DNA sampling regions (A). Individual admixture proportion of ancestry assigned to individuals in each of the K=2 subgroups is plotted for each individual (B). Individuals are ordered by DNA sampling region, and then by model-based likelihood (admixture proportion) for Cluster 1 for the Coastal BC (orange) and Haida Gwaii (red) sampling regions. For Interior BC (blue) and Alaska (purple) sampling regions, individuals are ordered by the likelihood for Cluster 2. (C) If an individual has a higher admixture proportion of belonging to Cluster 1 relative to Cluster 2 it is coloured yellow, if an individual has a higher admixture proportion for Cluster 2, it is coloured light blue (n=203, includes partial genotypes).


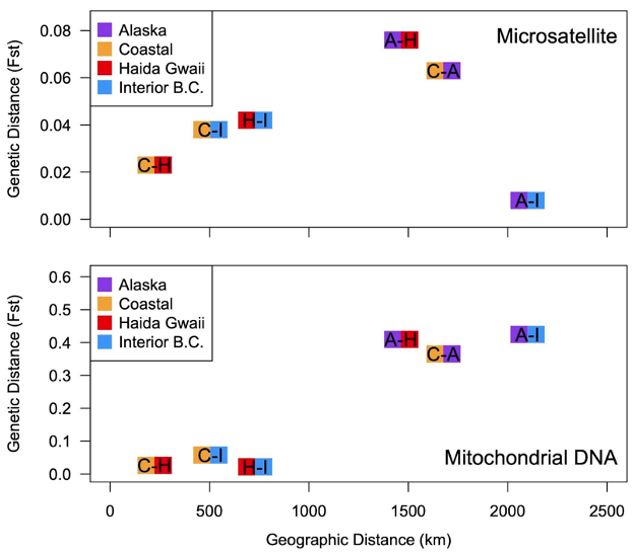


**Supplementary Figure** 3. Correlation between genetic distance (as judged by F_ST_) and geographic distance. The top panel shows this relationship for microsatellite data while the bottom panel shows the relationship for mtDNA. In both cases there is evidence of large genetic distances and large geographic distances between all populations and Alaska (n=203, includes partial genotypes).
